# Post-Translational Modifications of Lipoproteins: Emerging Players Linking Inflammation and Cardiovascular Disease in Rheumatoid Arthritis—A Narrative Review

**DOI:** 10.3390/ijms26178514

**Published:** 2025-09-02

**Authors:** Chuanhui Xu, Javier Rodríguez-Carrio, Yang Xie, Fanlei Hu, Wei Ming Chong, Han Wei Hou, Rinkoo Dalan, Khai Pang Leong

**Affiliations:** 1 Department of Rheumatology, Allergy and Immunology, Tan Tock Seng Hospital, 11 Jln Tan Tock Seng, Singapore 308433, Singapore; khai_pang_leong@ttsh.com.sg; 2 Lee Kong Chian School of Medicine, Nanyang Technological University, Singapore 308232, Singapore; wchong026@e.ntu.edu.sg (W.M.C.); hwhou@ntu.edu.sg (H.W.H.); rinkoo.dalan@nhghealth.com.sg (R.D.); 3 Area of Immunology, University of Oviedo, 33006 Oviedo, Spain; rodriguezcjavier@uniovi.es; 4 Area of Metabolism, Instituto de Investigación Sanitaria del Principado de Asturias (ISPA), 33011 Oviedo, Spain; 5 Department of Rheumatology and Immunology, Peking University People’s Hospital & Beijing Key Laboratory for Rheumatism Mechanism and Immune Diagnosis (BZ0135), Beijing 100044, China; xy1996_01_09@163.com (Y.X.); fanleihu@bjmu.edu.cn (F.H.); 6 State Key Laboratory of Natural and Biomimetic Drugs, School of Pharmaceutical Sciences, Peking University, Beijing 100081, China; 7 Department of Integration of Chinese and Western Medicine, School of Basic Medical Sciences, Peking University, Beijing 100081, China; 8 School of Mechanical and Aerospace Engineering, Nanyang Technological University, Singapore 308232, Singapore; 9 Department of Endocrinology, Tan Tock Seng Hospital, Singapore 308433, Singapore

**Keywords:** rheumatoid arthritis, post-translational modifications, inflammation, lipoproteins, cardiovascular disease

## Abstract

Patients with rheumatoid arthritis (RA) have an increased risk of cardiovascular disease (CVD) that cannot be fully explained by traditional cardiometabolic risk factors. The observed ‘lipid paradox’, where RA patients with lower total cholesterol and low-density lipoprotein cholesterol (LDL-C) levels exhibit higher CVD risk, may be attributed to post-translational modifications (PTMs). These lipoprotein PTMs likely arise from inflammatory pathways. While PTMs like citrullination and carbamylation are well recognized in RA joint pathology, their occurrence in other protein compartments and their role in CVD have been less well explored. This scoping review summarizes the current literature on PTMs of lipoproteins, including oxidation, nitration, carbamylation, and citrullination, and their impacts on CVD in RA. We also discuss immune responses to these PTMs, their interactions with scavenger receptors, and the effects of disease-modifying antirheumatic drugs. Further research on PTMs may uncover new pathways linking autoimmunity, inflammation, and vascular damage, offering novel diagnostic and therapeutic opportunities for RA-associated CVD.

## 1. Introduction

Rheumatoid arthritis (RA) is a prototypical chronic inflammatory disease with a prevalence of about 0.5% to 1% worldwide, characterized by symmetrical polyarthritis with multiple extra-articular manifestations [1]. Although significant advances have been achieved in the clinical management of RA, especially in terms of early diagnosis, the treat-to-target strategy, and the armamentarium of novel therapies, the incidence of cardiovascular disease (CVD) remains high and significantly contributes to mortality and morbidity [2]. In meta-analyses, both cardiovascular morbidity and mortality have been found to be 1.5-fold higher in RA compared to the general population [3,4]. In a review of 50 studies including 91,618 patients and 33,250 deaths, CVD accounted for 39.6% of mortality [5]. It has been reported that RA patients have a higher CVD risk than those with diabetes mellitus (DM) [6]. The young population is disproportionately affected by CVD, with incidence peaking in the third through fifth decades of life, causing substantial individual and societal burden [1,7,8]. Classical (“traditional”) CV risk factors in RA include male sex, age, hypertension, dyslipidemia, diabetes, smoking, and obesity. However, the increased incidence of vascular dysfunction, premature atherosclerosis, and CVD in RA is not fully explained by these traditional factors [9], and the elevated CVD risk persists even after adjusting for them [10]. Interestingly, 30% of the total CV risk can be attributed to RA characteristics, a figure comparable to that of total cholesterol or diabetes [11]. Moreover, it is important to note that RA itself has been reported to increase the risk of having CV risk factors as well [12]. Furthermore, 70% of CVD events were attributable to all CVD risk factors and RA characteristics combined [11], hence pointing to a significant proportion of the risk from unknown origin. As a consequence, traditional cardiometabolic risk factors alone fail to predict CVD in RA populations, hence emphasizing the need for additional tools. Framingham and the Systematic Coronary Risk Evaluation (SCORE) are commonly used to calculate the 10-year risk of CVD events in the general population [13,14], although their performance in RA patients is suboptimal [15,16], in part due to their sole reliance on traditional risk factors. The European Alliance of Associations for Rheumatology (EULAR) taskforce recommends adapting the general population CVD risk algorithms with a 1.5 multiplication factor for all patients with RA [9]. However, applying this multiplication factor does not reclassify all patients with RA into a more appropriate risk category [15,17,18]. CV risk prediction models incorporating an RA factor, such as the Expanded Cardiovascular Risk Prediction Score for RA (ERS-RA) and QRISK2, did not demonstrate superiority to the conventional scores either [16,19,20,21].

Understanding the underlying causes of this excess risk is instrumental not only to expand the knowledge in this area but also to identify potential biomarkers that may assist in CV risk stratification. The investigations on shared genetic components are conflicting. A recent genome-wide association study (GWAS) involving 26,637 Swedish patients with RA and controls without RA showed a minor genetic overlap between patients with RA and myocardial infarction [22], while a study using UK Biobank data supported the shared genetic pathogenesis between RA and CVD [23]. These contradictory results may be explained, at least in part, by differences in clinical endpoints and endpoint definitions, differences in analytical pipelines between studies, and potential differences in the role of traditional CV risk factors in RA and non-RA populations. Furthermore, RA patients present with a higher risk of CVD than those with DM [6]. Differences in pathogenic mechanisms underlying CVD between RA and the general population may also account for this heterogeneity in genetic overlap. Pathway analyses and polygenic risk scores are needed to gain insight into the relevance of these findings.

Evidence from genetic, pre-clinical, and clinical literature suggests that shared pathways exist between RA and CVD progression, where inflammation plays a pivotal role [24,25]. In this scenario, the interaction between inflammatory pathways and lipid metabolism is thought to drive CVD, although the exact components of this crosstalk are yet to be identified. A major postulate is that while common genetic substrates may be present, other phenomena, such as protein composition and post-translational modifications (PTMs) of lipoproteins, may act on top of genetic determinants in accounting for this excess risk. Accordingly, we examine the role of lipoprotein PTMs in this review.

PTMs encompass a group of chemical modifications, mostly by covalent irreversible changes, although reversible forms can also occur, which change the native structure of the proteins. PTMs can be derived from enzymatic or non-enzymatic reactions [26]. PTMs can be exhibited on circulating proteins, but recent evidence suggests that they can also be found on the surface of circulating extracellular vesicles (EVs) [27]. Regardless of their location, either by prolonged and/or enhanced exposure, PTMs of proteins may become susceptible to an immune response through recognizing these modified proteins [27].

### Study Rationale and Aims

PTMs have been extensively linked to RA pathogenesis, mainly in the case of citrullination of a limited number of protein targets (i.e., collagen, vimentin, alpha-enolase), which leads to specific humoral responses. Other forms of PTMs, including glycosylation, have been reported in several protein targets [26,28], including immunoglobulins, and linked to RA development and prognosis [29]. However, evidence in other protein compartments, such as lipoproteins, has received less attention. Similarly, how PTMs relate to other disease manifestations outside the joints, such as CVD development, has been largely unexplored. Therefore, the main goal of this literature review is to summarize the current knowledge on lipoprotein PTMs and their role in the interplay between RA and CVD. A literature search was performed using the following terms: rheumatoid arthritis, cardiovascular disease, post-translational modifications, scavenger receptors, and lipoproteins. The findings of this literature search are presented narratively and grouped into lipoprotein PTMs, the role of humoral responses, the role of scavenger receptors, and the effects of therapies.

## 2. Emerging Evidence for Post-Translational Modifications of Lipoproteins in Cardiovascular Diseases and Rheumatoid Arthritis

Dyslipidemia plays a fundamental role in CVD. There are six major lipoprotein particles classified by their apolipoprotein composition and physicochemical characteristics: chylomicrons and chylomicron remnants, very low-density lipoprotein (VLDL), intermediate-density lipoprotein (IDL), low-density lipoprotein (LDL), high-density lipoprotein (HDL), and lipoprotein(a) (Lp(a)). HDL exerts antiatherogenic effects, whereas all other lipoprotein particles are regarded as atherogenic. The atherogenic effects of LDL are mediated by LDL receptors and scavenger receptors expressed on vascular and immune cells. The downstream effects on monocytes and macrophages and the formation of foam cells are enhanced when exposed to an environment of increased inflammation and oxidative stress [30]. The anti-atherogenic properties of HDL consist of the removal of cholesterol from macrophages (termed macrophage cholesterol efflux), antioxidation, anti-inflammation, protection against thrombosis, and maintenance of endothelial function [31].

Relatively low concentrations of total cholesterol, LDL-C, and HDL-C are found during the active phase of RA. Of interest, an inverse relationship exists between lipid profiles and CVD risk in RA patients, where lower LDL-C concentrations have been associated with higher CVD risk than moderate levels. This phenomenon has been termed as the ‘lipid paradox’ [32]. It has been postulated to result from inflammatory pathways having a qualitative atherogenic impact on the lipoproteins [33]. However, although systemic inflammation has been demonstrated to account for a limited proportion of this effect [34], other mechanisms have yet to be elucidated.

**Figure 1:**
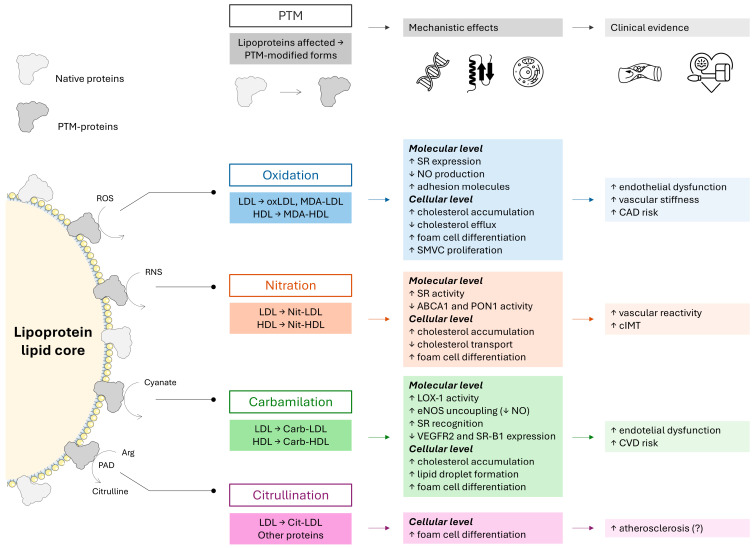
**PTM of lipoproteins.** Evidence of PTMs of lipoproteins is summarized according to the type of PTM, lipoproteins affected, effects at molecular and cellular levels, as well as associations with clinical and subclinical outcomes (see upper blocks). Upon certain stimuli, proteins contained in the lipoproteins (left) can change from their native conformation (light grey) to PTM-containing proteins (dark grey) (bottom).

In the “Progression of Early Subclinical Atherosclerosis” (PESA) study, half of the patients with subclinical atherosclerosis do not display abnormal LDL-C levels, suggesting that quantitative measurement of LDL-C is not sufficient to predict the development of atherosclerosis [35]. This adds to the notion of the ‘lipid paradox’ and urges the analysis of lipid profiles beyond mere quantification. Then, in this scenario, the role of PTMs is emerging. There is evidence demonstrating that the PTMs of lipoproteins are associated with the initiation and progression of atherosclerosis, both in the general population [36,37] as well as in RA [38,39]. In brief, PTMs of lipoproteins increase the proatherogenic effects of LDL-C and impair the antiatherogenic effects of HDL [36]. As a consequence, PTMs could be considered as promising novel non-traditional risk factors for CVD risk assessment, prevention, and therapy [37]. Some of these PTMs, such as citrullination, carbamylation, and glycosylation, are involved in the causation of both RA and atherosclerosis, extending the link between the two conditions, hence the concept of shared pathogenesis [28,38,39]. We will discuss the evidence for the role in CVD of RA of each type of PTM: oxidation, nitration, carbamylation, and citrullination (Figure 1).

### 2.1. Oxidation of Lipoproteins

**Figure 2:**
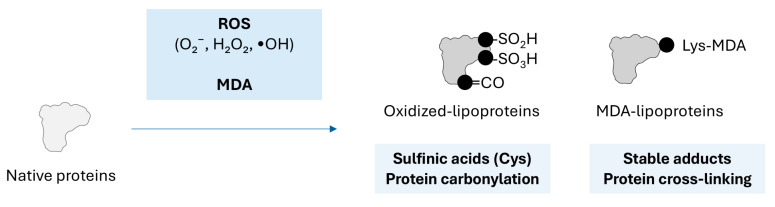
**Oxidation of proteins.** Schematic representation of oxidation reaction on proteins. Molecular mechanisms, chemical changes, and products are indicated.

In inflammatory states or oxidative stress, reactive oxygen species (ROS) are generated by cells. ROS are partially reduced derivatives of molecular oxygen. Key ROS include superoxide anion (O_2_^−^), hydrogen peroxide (H_2_O_2_), and hydroxyl radical (•OH) [40]. ROS are known to cause both reversible and irreversible protein modifications, mostly by the formation of sulfinic or sulfonic acids on Cys residues of the aminoacidic backbone, or by triggering protein carbonyls by oxidizing Lys, Arg, Pro, or Thr (Figure 2) [41,42]. These changes generally change the protein conformation and lead to altered function. Similarly, malondialdehyde (MDA) can react with the side chain of aminoacids in an additive manner, leading to changes in secondary structures, causing protein aggregation and altering functional properties (reviewed in [43]). ROS are implicated in the oxidative modification of LDL, transforming it into oxidized LDL (Ox-LDL). Oxidation of LDL is a gradual and continuous process, ranging from mild to extensive, encompassing different extents of various potentially toxic components of oxidized proteins and oxidized lipids. Thus, there is heterogeneity in the composition, metabolism, and biological characteristics of Ox-LDL particles.

Ox-LDL plays a vital role in the initiation and progression of atherosclerosis. Ox-LDL can upregulate the expression of scavenger receptors, thereby leading to cholesterol accumulation and foam cell formation [44]. It also provokes endothelial dysfunction, inhibits nitric oxide formation, and upregulates the expression of adhesion molecules that mediate the adhesion of neutrophils and monocytes, trans-endothelial migration, formation of foam cells, as well as phenotypic switching of vascular smooth muscle cells (VSMCs) [45]. The serum level of Ox-LDL is correlated with both plaque occurrence and instability [46]. Beyond atherosclerosis burden, it has also been found to be associated with arterial stiffness measured by carotid–femoral pulse wave velocity [47]. Ox-LDL is strongly associated with angiographically documented coronary artery disease (CAD) [48], and is a predictor for the development of CAD in healthy populations [49]. Although evidence is compelling overall, it is important to consider the heterogeneity of Ox-LDL formation [50,51], as well as the diversity of quantification protocols described in the literature (reviewed in [52]).

Ox-LDL concentration is significantly elevated in RA [53]. RA disease activity has been positively associated with Ox-LDL concentration and inversely with the total concentration of LDL and HDL [54]. This association was independent of other inflammatory markers [55]. An increased level of Ox-LDL predicts the subsequent CVD events in RA [56]. Similarly, MDA-modified LDL (MDA-LDL) particles have been reported to be increased in patients with RA and CAD, most significantly among RA patients with CAD [57]. Recently, a pilot randomized, double-blind placebo-controlled trial showed orticumab, a monoclonal antibody against MDA-LDL, inhibits coronary inflammation and reduces residual inflammatory risk in psoriasis, an inflammatory disease that has a similar CV risk burden as RA [58].

Recent evidence suggests a role for HDL oxidation in RA. Myeloperoxidase-mediated oxidation is known to impair the antiatherogenic effect of HDL on the cholesterol efflux capacity in RA [59]. Similar findings have been reported in diabetics [60,61], in line with the similarity in CVD figures between these two conditions [6]. Recent evidence has shed new light on other oxidative PTMs of lipoproteins, such as MDA-HDL in abdominal aortic aneurysm [62]. However, contrary to their LDL counterparts, there is no evidence in the literature about MDA-HDL levels in RA populations. The paucity of data on this, and potentially other modifications in HDL, poses important challenges to decipher the mechanistic underpinnings between inflammation, lipoprotein PTMs, and CVD in RA, as well as for their progression into clinical implementation. Therefore, further studies in this regard are warranted.

### 2.2. Nitration of Lipoproteins

**Figure 3:**
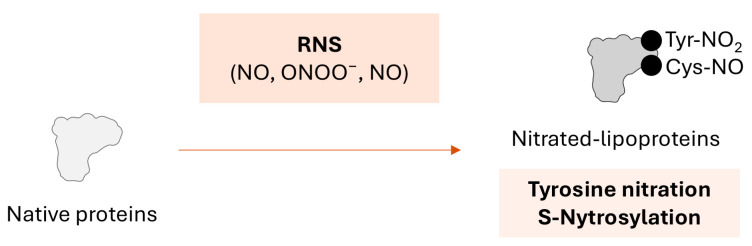
**Nitration of proteins.** Schematic representation of nitration reaction on proteins. Molecular mechanisms, chemical changes, and products are indicated.

Reactive nitrogen species (RNS) are derived from nitric oxide (NO), and include nitric oxide (NO), peroxynitrite (ONOO^−^), and nitrogen dioxide (NO_2_). Protein nitration involves the covalent addition of a nitro-containing group to a protein, most commonly on Tyr residues, but also on Trp, Cys, or Met (Figure 3). These changes lead to structural changes that can also trigger altered functionality or signaling mechanisms [63]. NO can react with superoxide to form peroxynitrite, a potent oxidant that can cause nitration and oxidation of lipoproteins [40]. The nitration of tyrosyl residues in the polypeptide chain of apoA-I and apoB results in the formation of nitrated lipoproteins, nitrated-LDL (Nit-LDL) and nitrated-HDL (Nit-HDL), respectively [64]. Nitrated lipoproteins have been associated with vascular reactivity and carotid artery intima-media thickness (cIMT) [65].

Nitration of apoB-LDL transforms LDL into a very potent ligand for macrophage scavenger receptors, particularly via the CD36 receptor. This contributes to cholesterol accumulation and foam cell formation [66]. Nit-LDL has also been found in atherosclerotic plaques, supporting the concept that this modification of LDL is important in the atherosclerotic process [67,68,69]. Increased concentrations of Nit-LDL in atherosclerotic plaques in CVD is independent of the presence of inflammatory disease [70].

Furthermore, it was demonstrated that the scavenger receptors are more sensitive to Nit-LDL compared to Ox-LDL [70]. Specific to patients with RA, there was no significant difference in levels of circulating Ox-LDL between patients with and without CVD when matched for comorbidity. However, levels of Ox-LDL protein were elevated when compared to young healthy controls. In contrast, Nit-LDL was significantly higher in RA patients with CVD compared with disease-matched controls and in osteoarthritis patients with CVD compared with disease-matched controls. In the absence of CVD, there was no significant difference between protein nitration in control subjects compared with patients with inflammatory disease [70].

Nit-HDL concentrations were also found to be higher in RA patients with CVD compared to their CVD-free counterparts. Despite the known anti-atherogenic properties of HDL, Nit-HDL was found to be related to a decrease in the activity of caspase-3, paraoxonase-1, and cholesterol transport via the ATP-binding cassette transporter A1 (ABCA1), and this decreases the CV benefits that are usually associated with HDL particles [71]. The presence of Nit-HDL is also associated with decreased cholesterol efflux and may be the contributory mechanism for atherosclerosis [59]. In one study, the concentration of Nit-HDL was also shown to be an early marker of microangiopathic dysfunction, assessed via the vascular reactivity index (VRI), among African American patients with type 2 diabetes, in the total population, and in subjects with HbA1c ≤ 7.0% [71].

However, the relationship between the concentration of nitrated lipoproteins and characteristics of subclinical cardiovascular dysfunction in RA is lacking.

### 2.3. Carbamylation of Lipoproteins

**Figure 4:**
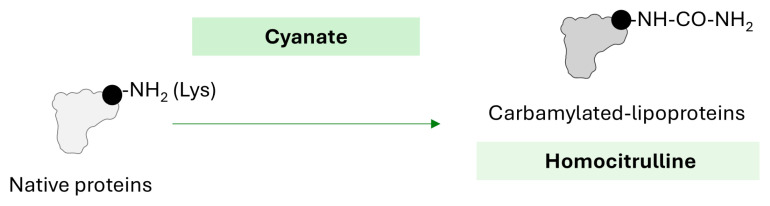
**Carbamylation of proteins.** Schematic representation of carbamylation reaction on proteins. Molecular mechanisms, chemical changes, and products are indicated.

Carbamylation is a form of PTM that is primarily induced by the presence of cyanate (isocyanic acid), a reactive nucleophile that irreversibly reacts with amino or sulfhydryl groups of amino acids by the addition of a carbamoyl moiety (-CONH_2_) to a functional group, usually on Lys residues, hence leading to ε-amino-carbamyllysine, also known as homocitrulline [72] (Figure 4). This process is not catalyzed by enzymes. Clinical studies have shown that carbamylated proteins (measured as plasma levels of protein-bound carbamyllysine) are independent risk factors for the development of CAD, myocardial infarction, and stroke in the general population [73]. Interestingly, recent evidence has revealed a significant correlation between autophagy and carbamylation in blood cells from RA patients [74], hence strengthening the role of autophagy. However, no evidence of the relationship between carbamylation and CVD has been reported in RA.

Carbamylated LDL (Carb-LDL) is the most abundant LDL modification found in human plasma [75]. Carb-LDL induces endothelial dysfunction through different mechanisms. One of these mechanisms is the activation of endothelial lectin-like-oxidized LDL receptor-1 (LOX-1), which impairs endothelium-dependent vasodilation [76]. Carb-LDL also stimulates the uncoupling of endothelial nitric oxide synthase (eNOS). This impairs NO production and triggers ROS instead, thereby inhibiting endothelium-dependent relaxation. Carb-LDL promotes the adhesion between monocytes and endothelial cells, and stimulates apoptosis of endothelial cells via the mitogen-activated protein kinase (MAPK) pathway [77]. Additionally, carbamylation leads to increased scavenger receptor recognition of LDL. Specifically, it was found that Carb-LDL is recognized by scavenger receptor class A type 1 (SR-A1) but not by scavenger receptor CD36 [73]. Overall, the increased recognition of Carb-LDL causes increased cholesterol accumulation in macrophages and foam cell formation, endothelial cell apoptosis, and VSMC proliferation [73]. These are all key processes that play a role in the development of atherosclerosis. As Carb-LDL has been shown to accumulate in endothelial and subendothelial layers of the aorta and heart [78], increased Carb-LDL levels are, hence, associated with a greater risk of CVD.

While HDL is commonly known to have anti-atherogenic properties, carbamylated HDL (Carb-HDL) has been found to play a role in CVD through several mechanisms. First, Carb-HDL suppresses the expression of vascular endothelial growth factor receptor-2 (VEGFR2). The VEGFR2 pathway is usually involved in migration, angiogenesis, and proliferation of endothelial cells after vascular injury [79]. Second, Carb-HDL also downregulates the scavenger receptor class B type I (SR-BI) signaling pathway in endothelial cells. SR-BI is a multiligand receptor that mediates HDL-induced endothelial cell migration, proliferation, and angiogenesis [80]. Hence, the suppression of both the VEGFR2 and SR-BI pathways results in increased risk of endothelial dysfunction and atherosclerosis. Third, Carb-HDL also induces lipid-droplet formation in macrophages. The balance between uptake and efflux of cholesterol is normally regulated by the interaction between Carb-HDL and SR-BI. Carbamylation disrupts this balance, causing a net cholesterol uptake [81]. The function of SR-BI varies depending on its location in the body. As SR-BI is especially upregulated in macrophages and in human atherosclerotic lesions of the aorta [73,82], HDL carbamylation can result in markedly increased cholesterol influx, thus contributing to atherosclerosis and ultimately leading to CVD burden.

Under physiological circumstances, cyanate levels are too low to cause a significant level of carbamylation. However, in inflammatory states like RA, myeloperoxidase (MPO) activity is elevated, and this can stimulate carbamylation [59,83]. MPO levels have been shown to correlate with disease activity in RA [84]. MPO enzymatically catalyzes carbamylation of LDL, converting it into a high-affinity ligand for SR-A1, especially at low levels of carbamylation [73]. In parallel, MPO-driven carbamylation of HDL impairs its vasoprotective functions, including its ability to prevent endothelial cell apoptosis and smooth muscle cell proliferation [73]. Thus, elevated MPO activity in RA promotes both enhanced LDL atherogenicity and reduced HDL functionality. Given that one of the mechanisms of action of statins includes the downregulation of MPO expression [85], suggesting MPO may serve as a modifiable therapeutic target for mitigating CVD risk in RA. Additionally, patients with RA have immune responses to carbamylated proteins/peptides [86]. These elevated levels of carbamylation can increase the atherosclerotic risk among RA patients, as carbamylated proteins and immune complexes have been detected in the joints of RA patients and in atherosclerotic plaques [73,87] (see below).

### 2.4. Citrullination of Lipoproteins

**Figure 5:**
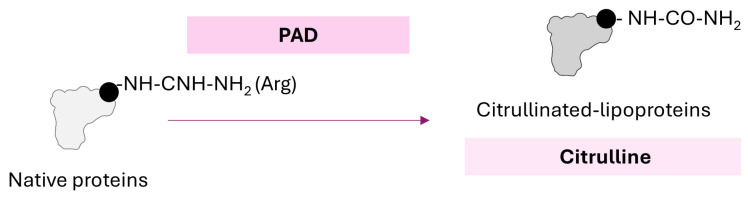
**Citrullination of proteins.** Schematic representation of citrullination reaction on proteins. Molecular mechanisms, chemical changes, and products are indicated.

Citrullination refers to enzymatic post-translational modification mediated by peptidyl arginine deiminases (PADs), which convert peptide-bound Arg residues into citrulline by eliminating an -NH_2_ group [88] (Figure 5). Structural similarities can be observed between citrulline and homocitrulline (from carbamylation), but underlying processes are largely different [38]. Citrullination leads to changes in protein charge (from positive to neutral) and loss of intermolecular interactions, proteolytic degradation, changes in protein conformation and unfolding, and ultimately altered function [89]. Interestingly, autophagy has also been linked to citrullination, and consistent evidence of PAD activation in autophagic cells has been demonstrated [90,91]. Importantly, a potential role has been suggested in antigen-presenting cells, hence linking autophagy, citrullination, and immune response in this scenario [92] (reviewed in [27]).

In RA, protein citrullination occurs at mucosal sites due to inflammation from smoking and/or microbial dysbiosis in genetically susceptible individuals, and this leads to T- and B-cell responses to citrullinated proteins/peptides [93]. In fact, the anti-citrullinated protein/peptides autoantibody (ACPA) was added to the classification criteria of RA in 2010 [94]. The presence of ACPA is associated with an increased risk of developing RA among healthy individuals and is detectable in healthy persons prior to the development of clinical RA. The antibody also has a prognostic role, as its presence is associated with more severe structural damage, radiographic progression, and poorer response to therapy [95]. Moreover, the role of citrullination extends beyond its implications for RA pathogenesis and prognosis, influencing cardiovascular risk in RA patients [96].

The presence of ACPA is significantly associated with the risk of CVD in RA in some [97], but not other studies [11,15,98]. However, citrullinated proteins are found in atherosclerotic tissues in patients with RA [39], and ACPA may drive some cardiometabolic mechanisms that promote CVD [99]. Collectively, these lines of evidence suggest that, although ACPA may not be a candidate biomarker for risk stratification, citrullination may play a determinant role in the progression of atherosclerosis, implying a role in pathogenesis. More importantly, deep characterization of ACPA specificity may be crucial to understanding whether joint-specific citrullination patterns mirror those of atherosclerotic plaques and lipoprotein antigens.

Studies on citrullinated lipoprotein are scarce. Citrullinated LDL (Cit-LDL) artificially induced by PAD2 could promote foam cell formation in human primary macrophages and has been found to be equally potent compared to carbamylated LDL [100]. However, Cit-LDL was not detected in RA patients or healthy controls in this study. It was postulated that the degree of modified amino acids in Cit-LDL is low. This is worth further investigation with more sensitive detection methods. The role of Cit-LDL and Cit-HDL in CVD in RA warrants further investigation.

## 3. Humoral Immune Responses Against PTMs of Lipoproteins

Atherosclerosis is an inflammatory disease with infiltration of inflammatory cells, including T and B cells [101]. Importantly, through the production of antibodies, humoral responses can lead to the activation of several populations and mechanisms within the immune system, which reinforces the promising relevance of this feature in the context of a complex phenomenon like atherosclerosis development. The role of the humoral immune response is well established in the pathogenesis of both RA and atherosclerosis, hence strengthening their shared pathogenic pathways [102]. PTMs can be exhibited on circulating proteins, but recent evidence suggests that they can also be found on the surface of circulating EVs [27], which contribute to cellular communication and signaling [103], including the transport of autoantigens and mediators between distant cells within affected tissues [104], MHC-mediated antigen presentation, and relevant processes such as adhesion molecule expression, endothelial activation, or pro-inflammatory pathways (reviewed in [105]), thus prompting specific humoral responses [27]. In fact, autoantibodies against protein-containing PTMs with EVs have been reported in RA populations linked to disease features [106]. Regardless of their location, either by prolonged and/or enhanced exposure, PTMs of proteins may become susceptible to an immune response by recognizing these modified proteins [27].

However, less evidence is available on how these mechanisms could affect atherosclerosis occurrence and progression. Although an initial focus was made on RA-related autoantibodies, i.e., rheumatoid factor (RF) and ACPA, more recent evidence from clinical studies has challenged this view [16,21,98]. More recently, anti-carbamylated protein (anti-CarP) autoantibodies have been found in the early phase of RA [38] and were previously identified as a predictor marker for the development of RA [107]. Not only are the levels of these autoantibodies significantly higher in patients with RA [108], but they are also associated with poor disease outcomes, including higher disease activity and radiographic progression [109,110,111] and mortality [112]. Similar to RF and ACPA, anti-carP antibodies have been reported to be associated with subclinical atherosclerosis measured as brachial artery Flow-Mediated Dilatation (FMD), cIMT, and arterial stiffness by the Ankle-Brachial Index and Cardio-Ankle Vascular Index in RA patients [113], although the confounding role of other autoantibodies and disease activity has yet to be considered. However, a previous study revealed that anti-Carb-LDL autoantibody was found in 18% of RA patients [100], hence suggesting not only that the process of carbamylation can be associated with an increased risk of CVD in RA but also strengthening the link among PTMs, humoral responses, and CVD. Taken together, the evidence highlights that although humoral responses may be related to CVD in RA, other specificities different than those targeted by disease-related autoantibodies may be involved.

**Figure 6:**
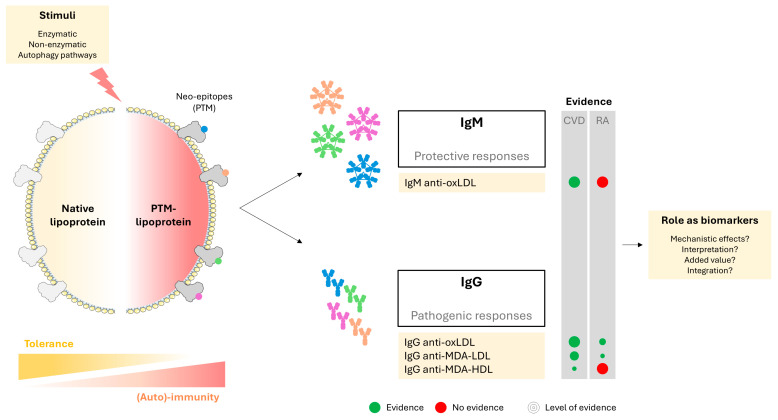
**Humoral responses against lipoproteins.** Although native lipoproteins are overall tolerated by the immune system, PTM-containing lipoproteins can elicit humoral responses mostly by the occurrence of neo-antigens. The different humoral responses documented in the literature are shown, and evidence in CVD and RA studies is depicted (left), indicating the presence (green circles) or absence (red circles) of published studies. Size of the circles represents the level of evidence.

In this scenario, the role of lipoproteins as autoantigens recently emerged, as several groups reported that autoantibodies targeting different lipoproteins, especially LDL and HDL, can be detected in RA. Moreover, changes and modifications in the protein cargo of lipoproteins have been reported [31], which may generate neo-epitopes that may elicit specific humoral responses. In general, it is widely accepted that B1 cells are atheroprotective through the production of IgM (natural) antibodies that may scavenge and/or impair the ability of PTM-modified lipoproteins to develop their pathogenic effects [114]. On the contrary, B2 cells are usually considered as atherogenic due to the production of atherogenic IgG and inflammatory cytokines, activating T cells and participating in antigen presentation as well (Figure 6) [102]. Furthermore, the production of IgG antibodies requires T-cell collaboration to engage in class switch, hence involving cognate T cells, namely follicular helper T cells, in this scenario. This is relevant in a twofold manner, as modulating T-cell/B-cell interaction may be helpful not only in understanding the atheroprotective/proatherogenic effects of the antibodies [115] but also represents a relevant therapeutic target [116]. Cytokines have been widely implicated in this crosstalk, especially IL-21 (by supporting B-cell proliferation and differentiation into germinal center B cells and plasma cells), Th1 and Th2 cytokines (by modulating isotype class switch), and IL-2 (by modulating antibody production) (reviewed in [115]). Furthermore, B-cell factors such as BAFF, BCMA, APRIL, and TACI play also relevant roles by shaping and maintaining the B-cell compartment, including the survival of memory B cells [115,117]. Importantly, recent data challenge the current dogma of IgM/B1 as atheroprotective and IgG/B2 as proatherogenic after the characterization of a novel autoantigen that can be recognized by IgG antibodies, which was demonstrated to delay and diminish atherosclerosis occurrence in animal models [118]. However, whether this also applies to RA and rheumatic diseases remains to be investigated.

As opposed to other conditions such as myocardial infarction [117], scant data is available on humoral responses against PTM-modified lipoproteins in RA (Figure 6), and it is mostly focused on oxidative PTMs. Early studies reported the development of antibodies against Ox-LDL [119]. Further studies confirmed these findings and showed that these antibodies could be associated with disease severity. Although some researchers found associations with atherosclerosis burden [120], conflicting results have been published with regard to CVD history [121]. These antibodies have been also linked with an increased incidence of vascular dysfunction and cardiovascular deconditioning in other immune-mediated conditions [122,123]. Compelling evidence may suggest that IgM antibodies against Ox-LDL particles belong to the oxidation-specific epitopes (OSE) [124], which are targeted by protective antibodies produced by B1 cells [125]. A similar picture may be observed for MDA-modified neo-antigens (see below). These antibodies are widely recognized as protective, both in the general population [126,127] as well as in rheumatic conditions [128,129]. Conversely, IgG antibodies, which are produced after T-cell collaboration with B2 cells, have harmful effects [122]. However, recent evidence has challenged this notion, hence demonstrating that marginal zone B cells can also produce natural (protective) antibodies in a T-cell-dependent manner [130], hence strengthening the role of T cells in this scenario. Interestingly, anti-Ox-LDL antibodies seem to underlie the association between low LDL-C level and atherosclerosis burden, possibly explaining the lipid paradox in RA [131]. However, controversial results were published thereafter [131,132], hence suggesting that their effects may be disease-specific or dependent of other (auto-)antibodies as well as on the PTM-modified lipoprotein levels.

MDA-LDL has also been reported to elicit a specific IgG humoral response in RA [121], and anti-MDA-LDL antibodies were found to be increased in patients with previous myocardial infarction, though they were not associated with other CVD outcomes. The level of LDL immunocomplexes predicted MDA-LDL levels in patients, along with systemic inflammation, hence suggesting that humoral responses against modified LDL particles may be a missing link among lipoproteins, inflammation, and CVD in RA. Although MDA-HDL particles are known to also elicit specific IgG humoral responses in mouse models and human individuals [133], whether these antibodies can be detected in RA populations has not been investigated yet.

Notably, despite some studies having addressed the differential effects of IgM and IgG antibodies, little is known about other isotypes (such as IgA or IgE) [134]. The same applies to IgG subclasses [124]. As effector functions and affinity for receptors located in cell types within the immune system and other tissues are largely dependent on these features, a deeper characterization may be useful in understanding the effects of these antibodies as well as their potential clinical relevance.

**Figure 7:**
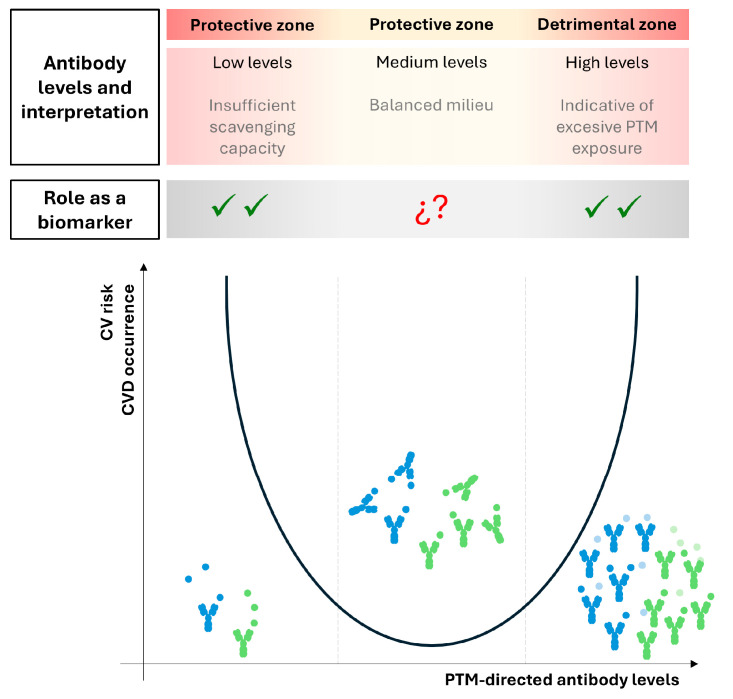
**Proposed model for the association between humoral response to PTM-lipoproteins and CV risk.** A conceptual model to explain the potential association between antibodies directed against PTM-lipoproteins and CVD risk is depicted. The U-shaped curve model suggests higher CVD occurrence both at low and high levels of antibodies, although underlying causes differ (top square), which may influence, and probably limit, their use as a biomarker (tick represent available evidence, question marks represent unclear/unavailable evidence). This model should be considered as a generalization, and it does not derive from the literature.

Humoral responses have important advantages as a source of clinical biomarkers, since their levels are relatively stable, which provide important advantages on pre-analytical, analytical, and clinical domains. Antibodies are relatively easy to measure in clinical practice. However, interpreting levels of antibodies against PTM-modified lipoproteins may be challenging for a number of reasons. First, it is difficult to differentiate whether the humoral responses are increased due to an active underlying ongoing immune response or are the consequence of an amplification due to the existing immune activation in the context of a chronic autoimmune condition. Second, increased antibody levels, regardless of their isotype, may exert a favorable effect in their role as scavengers (which is protective), but at the same time there are surrogate markers of the presence of PTM-lipoproteins (which are always deleterious). Therefore, it is tempting to speculate that the association between humoral responses and CVD may rather follow U-shaped curves, with higher risk being observed at both extremes (as low antibody levels may limit the scavenging of modified lipoproteins, and higher levels may be indicative of a higher lipoprotein modification) (Figure 7), thus posing an additional layer of complexity for implementation in clinical practice. Finally, despite extensive efforts in recent years, it is unclear how to better integrate novel biomarkers, such as antibody responses, into existing algorithms [135,136]. However, both modified proteins and antibodies against (native) lipoproteins have shown considerable promise in improving risk stratification [137,138,139]. Taken together, a joint assessment of PTM-lipoproteins and their humoral responses will be needed to better understand the role of humoral responses against PTM-lipoproteins, especially regarding their potential application as biomarkers.

## 4. The Synergistic Effects of Scavenger Receptors and PTMs of LDL on Atherosclerosis

**Figure 8:**
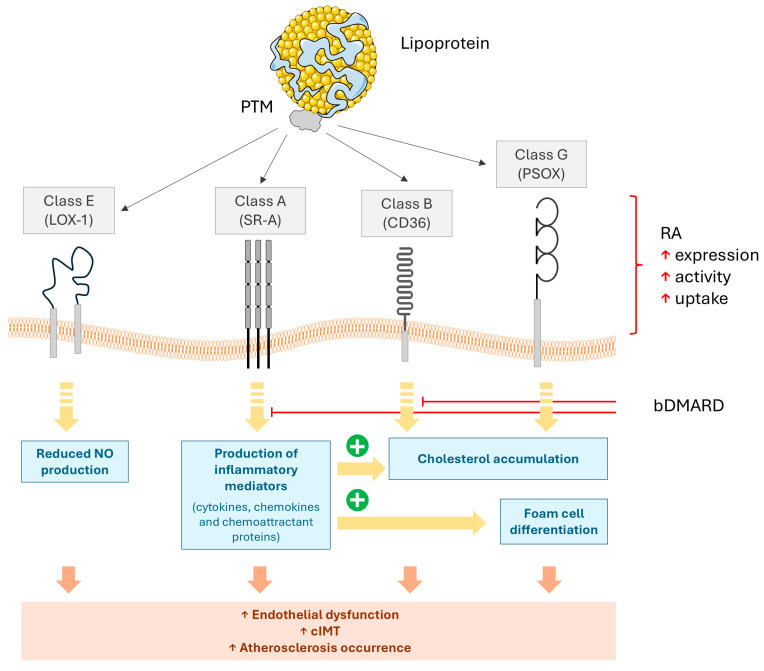
**Interactions between scavenger receptors and PTM-lipoproteins.** PTM-lipoproteins can be recognized by several scavenger receptors members. Main scavenger receptors classes (grey), downstream effects (blue), as well as clinical associations (orange) are depicted.

Scavenger receptors were initially defined by Brown and Goldstein in 1979, demonstrating their ability to bind Ox-LDL [140,141]. Accumulating evidence has shown that scavenger receptors are involved in atherosclerosis through Ox-LDL uptake into macrophages and promoting foam cell differentiation [142]. Scavenger receptors, as pattern recognition receptors, could also bind to expanding numbers of cognate ligands, such as native proteins, modified proteins, lipids, and polysaccharides, playing a role in cancer [143], Alzheimer’s disease [144], type 2 diabetes [145], and autoimmune diseases [146], etc. Different classes of scavenger receptors have been investigated in atherosclerosis, including class A: SR-A (scavenger receptor A), MARCO (macrophage receptor with collagenous structure); class B: SR-BI, CD36; class E: LOX1 (lectin-like oxidized LDL receptor 1); and class G: SR-PSOX (scavenger receptor for phosphatidylserine and oxidized low-density lipoprotein) [147] (Figure 8). Although other classes have been described (such as classes C, H, I, or J), no evidence of their connection with PTMs has been documented.

SR-A is predominantly expressed in macrophages, VSMCs, and endothelial cells. Upon binding Ox-LDL or acetylated LDL (Ac-LDL), SR-A activates NF-κB and MAPK signaling pathways, promoting the release of pro-inflammatory cytokines (i.e., TNF-α and IL-6) and the formation of foam cells [148]. CD36, another critical scavenger receptor, is highly expressed in macrophages, adipocytes, and platelets. It recognizes ox-LDL and advanced glycation end-product-modified LDL (AGE-LDL), thereby initiating TLR4/NF-κB signaling and PPARγ-mediated lipid accumulation, establishing a link between metabolic disorders and plaque instability [149]. LOX-1, primarily expressed in endothelial cells and macrophages, binds to Ox-LDL and C-reactive protein (CRP), activating ROS-dependent NF-κB signaling. This interaction results in endothelial dysfunction and an elevated risk of thrombosis [150]. In contrast, scavenger receptor class B type I (SR-BI) is predominantly expressed in hepatocytes and steroidogenic cells, where it facilitates the uptake of HDL and enhances reverse cholesterol transport, emerging as a therapeutic target to reduce atherosclerosis development [151]. Nevertheless, under conditions of elevated Ox-LDL, SR-BI may exhibit pro-atherogenic effects [152].

The tissue-specific distribution of these receptors critically influences their pathological roles in atherosclerosis: SR-A and CD36 in macrophages contribute to foam cell formation, LOX-1 in endothelial cells upregulates adhesion molecules such as VCAM-1 and ICAM-1 to recruit monocytes, and SR-A in VSMCs promotes fibrous cap disruption [153]. These downstream effects include sustained inflammation, characterized by the secretion of interleukin (IL)-1β (IL-1β) and IL-6, expansion of the necrotic core, and increased plaque vulnerability.

Scavenger receptors have also been demonstrated to play a role in autoimmune diseases, especially RA. For instance, SR-A was demonstrated to accelerate the onset of arthritis in collagen-induced arthritis (CIA) mice, and inhibition of SR-A by SR-A-neutralizing antibody and antagonistic ligand could ameliorate the disease severity [154]. Then, it is possible that the scavenger receptors and certain PTMs of lipoproteins may synergistically promote premature atherosclerosis in RA. Scavenger receptors are involved in atherosclerosis by uptake of Ox-LDL into macrophages and promoting foam cell differentiation (Figure 8). They also play a role in RA pathogenesis, suggesting that scavenger receptors may link inflammation and CVD by sensing PTMs of lipoproteins in RA.

Compared to healthy subjects, the serum from RA could induce overexpression of SR-A in human arterial endothelial cells, which was abrogated by the blockade of receptors of IL-6 or tumor necrosis factor (TNF)-α [155]. Ox-LDL-induced monocyte chemoattractant protein (MCP)-1 production can be augmented by IL-6 or TNF-α, as well as by the serum from RA patients. This augmentation could be partially reduced by blockade of receptors of IL-6 or TNF-α [155]. Taken together, the findings reinforce the role of SR-A and MCP-1 in endothelial dysfunction in RA and warrant further studies in relation to the therapeutic exploitation of this interaction under biological DMARD usage.

CD36 has been indicated to have a role in cardiovascular complications in RA through internalizing Ox-LDL. Wen et al. demonstrated that serum in CIA mice could significantly increase the expression of CD36 in murine macrophages [156]. The upregulation of CD36 and cholesterol accumulation in macrophages are likely mediated by decreased HDL-C and increased Ox-LDL, which can be reversed by simvastatin [156]. In contrast, the surface expression of CD36 was downregulated in peripheral blood mononuclear cells in RA patients with subclinical atherosclerosis compared with RA patients with normal cIMT [157]. Furthermore, Boyer et al. observed that the expression of CD36 was reduced by TNF-α and increased by adalimumab, a TNF-α inhibitor. Whether the dual function of CD36 was due to different pathways should be further corroborated.

As a class E scavenger receptor, LOX-1 is one of the major receptors of Ox-LDL, which was demonstrated as a novel biomarker for diagnostic and potential therapeutic targets in RA [158]. In zymosan-induced arthritis (ZIA) mice, the level of LOX-1 was accompanied by an increasing level of Ox-LDL [159]. In addition to its involvement in bone destruction in ZIA, the interaction between Ox-LDL and LOX-1 was reported to participate in endothelial dysfunction in RA [160]. In detail, through a human-like mice model of RA with transgenic activation of the TNF-α pathway, it has been shown that an increased level of TNF-α in RA could augment Ox-LDL uptake and activate the LOX-1/NFκB/Arg2 pathway, which reduced bioavailability of NO and levels of cGMP [160], hence leading to endothelial dysfunction.

CXCL16/SR-PSOX, a class G scavenger receptor, is upregulated in human myeloid leukemia mononuclear cells (THP-1) when incubated in plasma from RA patients, with increased uptake of Ox-LDL and subsequent formation of foam cells [161]. Interestingly, soluble CXCL16 was not correlated with plasma Ox-LDL and the presence of CVD in RA patients [162]. It is still unclear whether circulating CXCL16 might play a role in the balance of Ox-LDL uptake and degradation.

Despite not falling into the definition of SR, recent evidence suggests that a different class of receptors, termed low-density Lipoprotein Receptor-related Proteins (LRPs), play crucial roles in lipoprotein endocytosis [153]. Previous studies have found out that LRP6 and LRP8 have been involved in autoimmune diseases, namely antiphospholipid syndrome [163,164] and myasthenia gravis [165]. Evidence from genetic studies is supportive of the role of LRP5 in RA susceptibility and progression [166,167]. Additionally, antibodies against LRP2 have been described in RA patients, hence supporting its role as an autoantigen [168]. Further research is needed to understand better how LRPs impact lipoprotein metabolism in RA and whether their activation may be modulated by PTMs.

Collectively, these results suggest the involvement of the interactions between Ox-LDL and scavenger receptors with inflammatory circuits playing a major role in the pathogenesis of CVD in RA. However, besides Ox-LDL, the mechanisms of other PTMs of LDL and more clinical evidence need further study. Similarly, how LRP signaling may interact with these circuits remains unknown. Furthermore, whether this crosstalk could be manipulated for therapeutic purposes to alleviate CVD risk burden in RA has yet to be elucidated.

## 5. The Impacts of Disease-Modifying Anti-Rheumatic Drugs (DMARDs) on the Interplay Between Inflammation and Lipoprotein PTMs

Comorbidities are central to the holistic care of patients. Comorbidities, such as dyslipidemia, diabetes, or CVD, could impact the choice of treatment options. Importantly, some disease-modifying anti-rheumatic drugs (DMARDs) may also in turn modulate, either alleviating or aggravating, the comorbidity burden in RA. Accumulated evidence shows the reversal of the ‘lipid paradox’ with effective treatment of DMARDs in RA, although patients with similar degrees of disease activity control, and thus similar levels of immunosuppression, may exhibit important differences in lipid levels [33,169], thus suggesting the involvement of specific immune circuits in the maintenance of lipoprotein metabolism [33]. However, there is sparse data on the effects of qualitative changes of lipid profiles by DMARDs, namely PTMs of lipoproteins. Methotrexate provides a substantial survival benefit in a prospective cohort study, largely contributed by cardiovascular mortality reduction, which was not observed in other conventional DMARDs [170]. However, the CV-protective effect was not observed in a large randomized controlled study with patients with atherosclerosis at high CV risk. [171] This suggests that the disease-specific mechanism of elevated CVD can be modulated by methotrexate in RA, but not in the general population with atherosclerosis. A meta-analysis showed TNF inhibitors reduced all CV events by 30% (relative ratio 0.70, 95% CI: 0.54–0.90) and major adverse CV events (MACEs) by 70% (relative ratio 0.30, 95% CI: 0.15–0.57). [172] It has been demonstrated that the impairment of the antioxidative capacity of HDL-C can be improved with TNF inhibitors [173,174]. However, whether this effect is contributed by the change of PTMs of HDL-C is unknown. Tocilizumab, a fully-humanized monoclonal antibody targeting IL-6 receptor α, has been reported to increase the circulating levels of total cholesterol, LDL cholesterol, and triglycerides [175]. The underlying mechanism of increased LDL is due to the downregulated expression of LDL receptors on hepatocytes by tocilizumab [176]. Hence, a randomized control trial, cardiovascular safety of tocilizumab versus etanercept in RA (designated the ENTRACTE trial), was conducted to examine the risk of MACEs [177]. The trial showed that the estimated hazard ratio was 1.05 (95% confidence interval 0.77–1.43) for the occurrence of MACEs in the tocilizumab group relative to the etanercept group, despite the expected elevation of circulating lipid profiles in the tocilizumab group. Of interest, no increases in Ox-LDL were observed under tocilizumab treatment compared to those with placebo in the MEASURE trial [178]. In an observational study, Ox-LDL and Lp(a) levels reduced significantly after 3 months of treatment with tocilizumab [179]. This highlights the importance of qualitative rather than quantitative lipid profiles for CV risks. Blockade of either IL-6 receptors or TNF could reduce the expression of SR-A and augmented Ox-LDL-induced MCP-1 production [155]. Recently, the Oral Rheumatoid Arthritis Trial (ORAL) Surveillance showed a higher risk of MACEs in patients with tofacitinib, a JAK inhibitor, compared to those with TNF inhibitor [180]. This study resulted in a black-box warning issued by the Food and Drug Administration (FDA), not just for tofacitinib but also for upadacitinib and baricitinib, due to the potential class effect. Since the JAK-STAT pathway is shared downstream with many cytokine receptors, including IL-6 receptors, the elevation of circulating lipids is observed in patients with JAK inhibitors as well as those with tocilizumab [181]. However, the effects of tofacitinib on qualitative changes of lipid profiles is not well studied, and its effects on immunothrombosis must be also taken into account, as it seems to be a major determinant in explaining JAKi-associated CV risk.

In conclusion, despite evidence showing that certain DMARDs influence lipoprotein profiles and cardiovascular risks, the relationship between these changes and actual cardiovascular risk remains unclear. It is plausible that lipoprotein PTMs may account for this interplay between inflammation and CVD in RA, but further research is essential to clarify their role and contributory weightage in total cardiovascular risk. Gaining an understanding of these interactions will be instrumental to inform changes in treatment decision making, ultimately benefiting CV risk management, comorbidity burden, and patients’ quality of life.

## 6. Future Perspectives

Lipoprotein PTMs production and signaling could be associated with CVD in RA (Table 1). However, lower evidence is observed for lipoprotein studies compared with PTMs of other proteins in RA. A similar picture can be observed in relation to antibody responses (Table 1) (Figure 6). Immune pathways may be the missing link in orchestrating the probable bidirectional crosstalk between CVD and RA, promoting and sustaining the elevated risk of atherosclerosis and CVD in RA. First, the PTMs of lipoproteins are mainly driven by the chronic inflammation of RA. Second, the formation of corresponding (auto)-antibodies may activate additional immune and inflammatory circuits that in turn lead to enhanced pathogenic responses, both at the joint and vascular levels. However, whether genetic and epigenetic mechanisms influence susceptibility to lipoprotein PTMs remains unclear. For example, it is unclear whether genetic polymorphisms in enzymes such as PAD affect enzyme activity or specificity toward substrates, potentially altering PTM patterns. Epigenetic modifications could hypothetically affect the turnover of modified lipoproteins through mechanisms such as proteasomal recycling, influencing their accumulation or clearance [182]. Although antibody repertoires are genetically determined, the genetic contribution to the initiation and maturation of humoral immune responses specifically targeting PTM-lipoproteins has not yet been established. Advanced pathway analyses and polygenic risk scores represent promising tools to elucidate the broader genetic architecture linking PTM-lipoproteins to CVD in RA. Moreover, scavenger receptors, which serve as receptors for PTMs of LDL, are upregulated in RA, which synergistically drive the pathophysiological process of premature atherosclerosis. The roles of other elements, such as LRP, need to be established, especially in view of their therapeutic potential. There is a potential role of certain DMARDs to abrogate these pathogenic pathways, thus alleviating CVD risk excess, although evidence is scarce. In addition to actionable therapeutic targets, the PTMs of lipoproteins could be potential disease-specific biomarkers for risk stratification of CVD, facilitating early and personalized CVD risk management in RA. Furthermore, the role of autophagy as a driver of PTMs is emerging, by virtue of the connection between autophagy, EVs secretion, and lipid metabolism [183]. Contemporary evidence suggests that therapeutic modulation of autophagy may control cholesterol metabolism and atherosclerosis [184]. Vesicles are also promising therapeutics, although the level of evidence is much lower [185]. It may be conceivable that autophagy induction may enhance both PTMs occurrence and antigen presentation, hence promoting immune responses in this scenario. However, recent findings have suggested that PTMs may also drive autophagy themselves [186], and autophagy machinery is subjected to regulation by PTMs as well [187], thus pointing towards a feedback loop between PTMs and autophagy.

Despite the considerable potential, the limited evidence and knowledge gaps limit the translation of lipoprotein PTMs into clinical routine (Table 2). The study of the effects on qualitative changes of lipid profiles by DMARDs is warranted to optimize the holistic care for patients with RA, maximizing treatment outcomes and avoiding unwanted effects. Moreover, a better comprehension of these circuits may not only be beneficial for RA populations but will also be applicable beyond rheumatology, hence impacting on human CVD and the general population, where the manipulation of inflammation as part of the therapeutic armamentarium is gaining momentum in recent decades. Further research into clinical added value of, the interpretation of lipoprotein PTMs, and their elicited humoral responses are warranted.

One of the areas that can be delved into further is the use of advanced imaging modalities to visualize and quantify lipoprotein PTMs in atherosclerotic plaques. Previous studies attempted the use of 124I conjugated to scavenger receptor CD68 for the detection of atherosclerotic plaques in mice. Preliminary data suggest the possibility of using CD68 as a molecular marker, as CD68 binds to Ox-LDL molecules and mediates their uptake [188]. Other possible targets for Ox-LDL in SPECT/CT imaging and Magnetic Resonance include LOX-1 probe, which binds specifically to a region with vulnerable plaque features that are rupture prone [189]. Another potential target would be the PET target probe, 64Cu-3H3-scFv, which targets the β2-glycoprotein/OxLDL complex, previously demonstrated to bind to lipid deposits in atherosclerotic lesions in a rabbit model of hyperlipidemia [190]. However, most existing literature addresses the use of advanced imaging to visualize only Ox-LDL, mostly on animal models. Currently, there are no imaging modalities available for other forms of PTM (including nitration, carbamylation, citrullination). Mass spectrometry and immunohistochemistry remain the most definitive approaches to detect other forms of PTMs in lipoproteins. Further research can be conducted in this area for imaging modalities to detect specific PTMs in lipoproteins and facilitate early detection and identification of cardiovascular risk.

## Figures and Tables

**Table 1:** Summary of evidence on PTMs.

	Lipoprotein PTMs in Human	PTM in RA Patients	Lipoprotein PTMs in RA Patients	Specific Humoral Responses
***Oxidation***	Ox-LDL MDA-LDL MDA-HDL	Collagen	Ox-LDL MDA-LDL	IgM anti-Ox-LDL IgG anti-Ox-LDL IgG anti-MDA-LDL IgG anti-MDA-HDL
***Nitration***	Nit-LDL Nit-HDL	Nil	Nit-LDL among RA with CVD Nit-HDL among RA with CVD	Nil
***Carbamylation***	Carb-LDL Carb-HDL	Vimentin Alpha-Enolase Collagen	Unclear	IgG anti-Carb-LDL
***Citrullination***	Unclear	Vimentin Alpha-Enolase Collagen	Nil	Nil
***Acetylation***	Nil	Vimentin	Nil	Nil

Data on the development of PTMs in human subjects in general and in RA patients, and descriptions of humoral responses (based on human studies) are summarized. Whereas evidence on PTMs in RA is substantial, scant data on PTMs of lipoproteins is available (especially regarding protein targets). The amount of data on humoral responses is rather limited overall. Abbreviations: PTMs—post-translational modifications; RA—rheumatoid arthritis; CVD—cardiovascular disease; Ox-LDL—oxidized low-density lipoprotein; MDA-LDL—malondialdehyde-modified low-density lipoprotein; MDA-HDL—malondialdehyde-modified high-density lipoprotein; Nit-LDL—nitrated low-density lipoprotein; Nit-HDL—nitrated high-density lipoprotein; Carb-LDL—carbamylated low-density lipoprotein; Carb-HDL—carbamylated high-density lipoprotein; IgM—immunoglobulin M; IgG—immunoglobulin G.

**Table 2:** Research agenda on lipoprotein PTMs in RA populations.

Domains	Research Priorities
*Methodological*	Gold standards to measure PTMs of lipoproteins in human samples (***) Sensitive methods to detect PTMs of lipoproteins in human samples, particularly for citrullinated lipoprotein (***) Robust methods to accurately quantify PTMs of lipoproteins in human samples (*) Experimental models to evaluate the effects of PTMs of lipoproteins at molecular and cellular levels (**) Use advanced imaging techniques to correlate PTMs of lipoproteins with plaque characteristics and the risk of plaque rupture (*)
*Basic/translational*	Effects of the different PTMs of lipoproteins on cell subsets other than foam cells (VSMC, endothelial cells, lymphocytes, adipocytes, etc.) (***) Impacts of the different PTMs of lipoproteins on protein cargo of the lipoproteins (HDL and LDL particles) (**) Effects of PTMs of lipoproteins (of LDL and HDL particles) on anti-atherogenic properties of HDL (***) Identification of B-cell populations linked to the production of antibodies targeting PTMs of lipoproteins (*) Investigating the predominant isotype classes and subclasses against PTMs of lipoproteins Effects of the different antibodies against PTMs of lipoproteins on immune cell subsets (lymphocytes and monocytes/macrophages) via FcR receptors (**) Effects of genetic and epigenetic determinants on the formation of PTMs of lipoproteins (*) Evaluation of the independent effects of circulating PTMs and EVs-containing PTMs (*) Investigating the genetic architecture of humoral immune responses against PTMs of lipoproteins (*)
*Clinical*	Effects of IgM antibodies against PTMs on CVD outcomes in RA populations (**) Associations between clinical and immunological features with IgG and IgM antibodies against PTMs in RA populations (**) Relevance of genetic determinants of PTMs of lipoproteins as potential biomarkers to guide patient stratification (*) Effects of DMARDs, especially bDMARDs and tsDMARDs, on the levels of PTMs of lipoproteins (***) Effects of the drugs described above on the levels of antibodies against PTMs of lipoproteins, B-cell subpopulations, and B-cell-related factors involved in humoral responses Impacts of drugs targeting autophagy pathways on lipoprotein PTMs, and correlation with clinical benefit (*) Usefulness, reclassification potential and cost-effectiveness assessments of PTMs of lipoproteins on CV risk stratification in RA populations (**) Data integration of PTMs of lipoproteins into existing algorithms (***) Targeting PTMs of lipoproteins and/or scavenger receptors as potential therapeutics to mitigate the adverse CV effects in RA (*)

A priority score is indicated for each topic, where ‘***’ represents the highest, ‘**’ represent medium, and ‘*’ represents the lowest priority. Priority scoring relates to the dependency of the given topic in order to expand knowledge, allow further research, or transform and improve clinical care, according to the judgement of the authors. Abbreviations: PTMs—post-translational modifications; RA—rheumatoid arthritis; VSMC—vascular smooth muscle cells; HDL—high-density lipoprotein; LDL—low-density lipoprotein; FcR—fragment crystallizable region receptor; EVs—extracellular vesicles; IgM—immunoglobulin M; IgG—immunoglobulin G; CVD—cardiovascular disease; DMARDs—disease-modifying anti-rheumatic drugs; bDMARDs—biologic disease-modifying anti-rheumatic drugs; tsDMARDs—targeted synthetic disease-modifying anti-rheumatic drugs; CV—cardiovascular.

## Data Availability

No new data was used for the research described in the article.
